# The Danish Ibbis Trials for Sickness Absentees with Common Mental Disorders: A Phase 4 Prospective Study Comparing Randomized Trial and Real-World Data

**DOI:** 10.5334/ijic.7562

**Published:** 2024-07-26

**Authors:** Andreas Hoff, Anders Bo Bojesen, Lene Falgaard Eplov

**Affiliations:** 1 Copenhagen Research Unit for Recovery, Mental Health Services, Capital Region Denmark, Copenhagen University Hospital, Hans Bogbinders Allé3, 3. sal, 2300 København S, Denmark

**Keywords:** integrated care, common mental disorders, vocational rehabilitation, depression, anxiety

## Abstract

**Introduction:**

In two randomized controlled trials (RCT) we tested the efficacy of a novel integrated vocational rehabilitation and mental healthcare intervention, coined INT, for sickness absentees with common mental disorders. The aim was to improve vocational outcomes compared to Service As Usual (SAU). Contrary to expectations, the delivered intervention caused worse outcomes within some diagnostic groups and some benefits in others. In this phase 4 study, we examined the effectiveness of the intervention in real-world practice.

**Method:**

In this prospective intervention study, we allocated adult sickness absentees with either depression, anxiety, or adjustment disorder to receive INT in a real-world setting in a Danish Municipality. We compared the vocational outcomes of this group to a matched group who received INT as a part of the RCTs, after randomization to the intervention group herein. Primary outcome was return to work at any point within 12 months.

**Results:**

In the real-world group, 151 participants received INT during 2019. From the randomized trials, 302 matched participants who received INT between 2016–2018 were included. On the primary outcome – return to work within 12 months – the real-word group fared worse (48.3 vs 64.6 %, OR 0.54 [95%CI: 0.37–0.79], p = 0.001). Across most other vocational outcomes, a similar pattern of statistically significant poorer outcomes in the real-world group was observed: Lower number of weeks in work and lower proportion in work at 12 months (42.3% vs. 58.3% (p = 0.002)).

**Discussion:**

The real-word group showed significantly worse vocational outcomes. Like in many other studies of complex interventions, implementation was difficult in the original randomized trials and perhaps even more difficult in the less structured real-world setting. Since the intervention was less effective for some groups compared to SAU in the original trial, this negative effect may be even more pronounced in a real-world setting.

## Introduction

Common mental disorders (CMD) like anxiety, depression, and stress-related disorders alone account for 40% of all sickness absence longer than eight weeks [1]. These disorders are associated with much suffering and large societal costs due to production loss and expenses for services and sick leave benefits. Furthermore, sick leave duration is associated with risk of permanent labour market exclusion [2]. For these reasons, much intervention research has focused on vocational outcomes, like the most frequently used being sick leave duration, but also proportion in work at follow-up is seen [3]. Many interventions have been studied with heterogenous results, but according to a comprehensive systematic review [4], the most effective interventions seem to be ones who:

- have more than one component, e.g. both psychotherapy *and* a work-focused intervention, yielding a complex intervention defined as those “made up of various interconnecting parts”, as defined by Campbell et al. [5]; furthermore they should
- focus on early, graded return to work; and finally they should
- emphasize workplace involvement [4].

The interplay between components in a complex intervention can take many forms, like e.g. interconnected as mentioned above, but it has also been suggested that they should be *integrated* [6], and another study of such integration of interventions showed positive results [7].

But such complex or integrated interventions are quite hard to deliver with high fidelity, even in settings of a randomized controlled trial (RCT), despite often detailed intervention protocol and rigorous attempts to achieve high protocol fidelity [8]. Adding further complexity, the value of RCT results depends on their generalizability to real-world settings [9], and if external validity is low due to even lower fidelity when the rigorousness of the RCT setting is changed to a real-world setting, the gap between intervention effect in these domains may be significant [10]. For that reason, there is a growing consensus that any intervention showing significant efficacy in an RCT should followingly be tested in a so-called “phase IV study” (referring to the RCT as the typical methodology in phase III studies), to determine intervention effectiveness, being an intervention’s effect in a real-world setting [11]. The study presented in this paper, is such a phase IV study, where we compare the effect of a complex intervention we trialed in a previous study, to the effect of the same intervention in a real-world setting. The principal intervention we test in this phase IV study, is an integrated intervention we previously tested in two randomized controlled trials (RCT), the IBBIS Trials [12,13].

### The original IBBIS Trials

**Figure 1:**
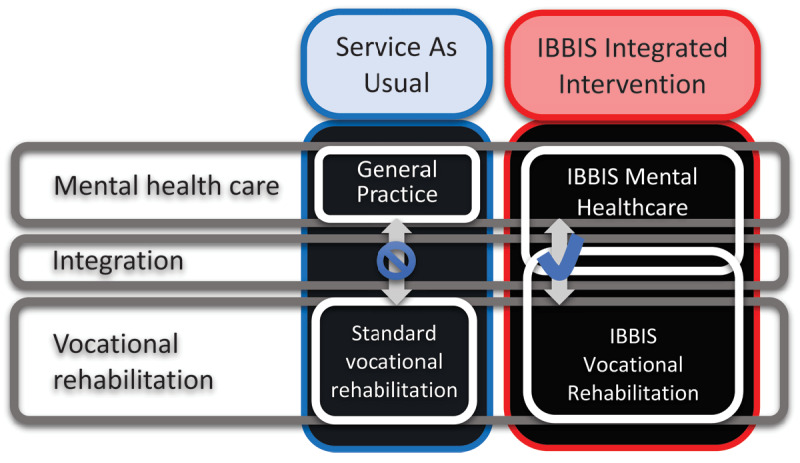
Composition of intervention components. “IBBIS” is a Danish acronym translating to “Integrated Health Care and Vocational Rehabilitation for Sick-Leave Benefit Recipients”.

“IBBIS” is a Danish acronym translating to “*Integrated Health Care and Vocational Rehabilitation for Sick-Leave Benefit Recipients*”. Both RCTs included adult participants on sick leave with common mental disorders, with an average baseline sick leave duration of 10 weeks (SD 4). One RCT included absentees with depression or anxiety as their main diagnosis (RCT1) and the other absentees with a stress-related disorder (RCT2). Both studied the effect of the IBBIS Integrated Intervention (INT) by comparing it to Service As Usual (SAU), which in Denmark consist of treatment in General Practice, combined with standard work rehabilitation intervention and case management in the local municipality, and these sector do not cooperate, but in some cases exchanges information unidirectionally from GPs to the municipality during sick-leave – see Figure 1 depicting SAU in left column. Both trials were pre-registered on ClinicalTrials.org and in two design papers [14,15].

### Results of the IBBIS Trials

In RCT1 (target group: anxiety and depression), we found that INT showed benefits regarding probability of being in full-time work at 12-month follow-up (a secondary outcome) but no effect on the primary outcome, return to work measured at 12-month follow-up [16]. In RCT2 (target group: stress-related disorders), INT consistently showed significantly worse vocational outcomes across different measures [17].

In both IBBIS Trials, implementation was suboptimal. A process evaluation study showed that diverging norms and goals between staff groups of the different sectors supposed to be integrated regarding norms and goals. It was concluded that that hindered the integration of the intervention components [18]. The intervention protocol stipulated that intervention components should imply goal *alignment* between the goals of the healthcare staff and the employment consultants delivering the vocational rehabilitation intervention, but instead a goal *hierarchy* gradually developed and settled during the two-year trial period, with the goals of the latter staff group dominated over the other [19]. These implementation issues constituted the largest challenges to the studies’ external validity. Either the implementation issues meant that the results of the trial could not be ascribed the true effects of the IBBIS Integrated Intervention, if it had been delivered, or either this intervention is so hard to deliver, even in a rigorous RCT setting that in the real-world it is unfeasible.

### Aim of this study

In accordance with the above discussion, a process evaluation of the IBBIS Trials led to the conclusion that a complex intervention like INT needs substantial managerial attention to be successfully implemented in a real-world setting [18]. After the inclusion to the IBBIS Trials was concluded, the Copenhagen Municipality decided to continue to deliver the IBBIS Integrated Intervention to the population of sickness absentees for which in was intended. This gave rise to the opportunity to study the effectiveness of the intervention in a real-world setting, and hence quantify any differential from the (negative) efficacy measured in the RCT setting. The aim was to compare the vocational outcomes of sickness absentees who received INT in the IBBIS RCTs with a comparable group of sickness absentees who received INT in a real-world setting INT (henceforth “real-world-INT”) after the RCTs ended.

## Methods

This study was registered before final analyses were performed. The preregistered statistical analysis plan can be found at https://osf.io/3ca5m/.

### Data sources

As in the original RCTs, we retrieved vocational information about the absentees from Danish national registers on social benefits and income (the Danish DREAM register). Information about diagnosis and start date of sick leave was collected by research staff. Covariate data on education, social benefit history and employment status was retrieved from Statistics Denmark.

### Recruitment procedure, eligibility assessment and inclusion

The study population in real-world-INT was recruited from the agency in Copenhagen Municipality, managing the cases of sick-leave benefiters. Recruitment procedure, and inclusion criteria was similar to the procedure in the IBBIS RCTs, except participants were not randomized, but offered inclusion in the intervention group if eligibility was established.

In RCT-INT, participants underwent a randomization procedure, before eligibility assessment, which again was conducted before receiving INT, whereas in the real-world-INT group, participants did not undergo randomization, but allocation to intervention if they were found eligible and gave written consent. Yet, in both groups, after recruitment to, and as a part of the eligibility assessment, participants a thorough mental health assessment, called the IBBIS Mental Health Assessment (IBBIS-MHA). IBBIS-MHA was performed by a psychiatrist or by a mental health professional (a nurse, psychologist, or psychiatric medical resident) supervised by a psychiatrist. The IBBIS-MHA consisted of a clinical interview with a focus on current mental health issues. The clinical interview started with a pragmatic clinical interview where the participants was asked about main health issues, and about what they experienced as main cause of sick leave. That part of the interview was followed by a) the semi-structured *MINI International Neuropsychiatric Interview* [20], to ensure a systematic approach to assessment of main mental health symptom domains; furthermore, to screen for personality disorders it was followed by the clinician-rated b) *Standardized Assessment of Personality – Abbreviated Scale* (SAPAS) [21] and c) *Attention deficit hyperactivity disorder symptom checklist for adults, Adult Self-Report Scale* (ASRS) [22]. The *Mini-Mental State Examination* (MMSE) was used if dementia was clinically suspected by the assessor [23]. Before the interview, participants filled in the validated Danish version of the *Four-Dimensional Symptom Questionnaire (4DSQ)* that measures levels of depression, anxiety, distress, and somatization [24]. Assessors had access to the results to guide their clinical assessment. We studied the isolated effects of the IBBIS-MHA in a separate study, since we could not rule out that this procedure could itself influence vocational outcomes. Hence, the outcomes in the SAU group in the IBBIS Trials may not totally reflect a true real-world-SAU, and the effect of IBBIS-MHA was needed to inform discussion of the external validity of the results of the IBBIS Trials. Due to methodological limitations, no strong conclusions could be drawn from that study, but we saw tendencies towards a negative effect on vocational outcomes from this assessment per se [25]. Before study inclusion, participants in RCT-INT filled in self-report questionnaires that participants in real-world-INT did not, but this data was not available to the assessors, solely used for study purposes in the RCTs why they where not utilized in this study.

#### Eligibility criteria

To be eligible for inclusion in the study groups participants had to be at least 18 years of age, be on sick leave for at least 4 consecutive weeks, have undergone a mental health assessment with a primary diagnosis of either anxiety, depression, or an adjustment disorder, including exhaustion disorder [26] or stress according to the Four-Dimensional Symptom Questionnaire [24].

### Study intervention: The IBBIS Integrated Intervention (INT)

Both study groups received INT, the IBBIS Integrated Intervention, yet in RCT- versus real-world- settings, respectively. The IBBIS Integrated Intervention (INT), consists of IBBIS Mental Healthcare and IBBIS Vocational Rehabilitation, and these two intervention components were integrated – see Figure 1, right column.

INT consisted of the integration of IBBIS Vocational Rehabilitation and IBBIS Mental Healthcare. These intervention components and the methods for integration are described in detail in the RCT study design papers [14,15]. IBBIS Mental Healthcare was a stepped-care intervention with treatment options depending on diagnosis. For participants with stress-related disorders, treatment included predominantly clinical monitoring, stress-coaching or, for those with exhaustion disorders, mindfulness-based stress-reduction (MBSR). Treatment of participants with anxiety or depression adhered to the guidelines from National Institute of Clinical Excellence [27], consisting primarily of psychoeducation, clinical monitoring, psychotherapy, medication or, in more severe cases, a combination hereof. Treatment was delivered by care managers, who were mental healthcare professionals with at least one year of experience and training in general mental healthcare and psychotherapy. IBBIS Vocational Rehabilitation was based on the principles of the *Sharp-at work* intervention [28] and *Individual Placement and Support* [29]. The vocational rehabilitation intervention components were provided by employment consultants, who also functioned as the case-managers of the participants sick-leave benefit cases.

These two principal intervention components, IBBIS Mental Healthcare and IBBIS Vocational Rehabilitation, were sought integrated through the following activities: I) co-location of staff; II) at least one roundtable-meeting between the care manager, the employment consultant, and each participant; and III) interdisciplinary training and supervision for care managers and employment consultants.

#### Protocol adherence in RCT-INT

In RCT-INT, staff adherence to the study intervention manuals was examined through fidelity interviews. While IBBIS Mental Healthcare was implemented with high fidelity, IBBIS Vocational Rehabilitation and the activities to ensure integration were only implemented with fair fidelity. For example, a central intervention component activity like work contact was only delivered to rather few participants. We saw a slight tendency of increasing fidelity over time during the study period, but we did not perform any statistical test.

### Control group selection

The group of study participants in the control group, RCT-INT, was a selected subset of participants from the group of participants randomized to INT in the original IBBIS Trials (RCT-INT).

We could not rule out that there were differences between group who were referred for eligibility assessment for an RCT, and a group referred to the same intervention in a less rigorous real-world setting. Therefore, through propensity score matching [30], we selected two controls per participant in the intervention group. Propensity score matching is a statistical method for finding the most similar participants(s) per one case, when several variables are available, and no exact match can be found, but one wish to find the most similar other case(s). Matching variables were age, sex, primary diagnosis (as established during the IBBIS Mental Health Assessment), employment status (with or without a current employment), work branch category (as defined by Statistics Denmark, e.g. *farming, production, teaching, administration* etc.), and social benefit history (how many weeks during the two years before baseline had the participant received social benefit payments). These co-variates were selected as matching variables since they are likely to influence vocational outcomes [31]. The general employment rate is also known to impact vocational outcomes among sickness absentees but could not be included as a matching variable; employment rates in society at the time of observation differed between the groups, as the groups received the interventions at different points in time between which the level of employment in society changed (before and after mid-2018, respectively). Crude employment rates are reported without any adjustment since such adjustment would entail assumptions about the exact causal effect of specific changes in employment rates on the vocational outcomes of the study population. Post-hoc, we conducted sensitivity analyses using municipality as a matching variable, since municipality was also associated with vocational outcomes in the RCTs. However, this halved the sample size of the RCT-INT group, as real-world-INT was only delivered in one of the municipalities involved in the RCTs. For that reason, municipality was not a matching variable in the main analyses.

### Outcomes

The primary outcome of this study was any return to work any time before 12-month follow-up. We chose this outcome because of its higher sensitivity compared to the more commonly used time-to-event analyses [3] and because, in one of the RCTs, INT seemed to have effect around 12-month rather than 6-month follow-up [32]. Return to work was defined as consecutive period of at least four weeks in which the participant did not receive any social benefit, like sick-leave benefit, and in which period the participant earned a salary through working in a competitive ordinary job. Two secondary outcomes were in a similar fashion any return to work but measured at 6- and 24-month follow-up, respectively. Further secondary outcomes, were, at 6-, 12-, and 24-month follow-up, respectively, proportion in work at follow-up, and number of weeks in work between baseline and follow-up.

### Statistical Analyses

Sample size was not determined through calculations, since the cohort size was determined externally, but before analyses were conducted, we calculated that with the number of participants in the study groups, power would be at least 0.8, for a difference of at least 13%-point between groups, since we assumed proportion of positive cases on the primary outcome would be between 0.2 and 0.65 (as observed in the original IBBIS Trials). We applied the intention-to-treat principles in all analyses. Due to the comprehensive nature of the Danish registers, we did not expect missing data and thus did not plan for this eventuality. Treatment effects were estimated using logistic regression for binary outcomes, including the primary outcome. Poisson regression with bootstrapped non-parametric standard errors was used for numeric outcomes, and 1000 resamples were applied. A significance level of 5% in two-sided tests was used throughout. This applied to p-values and all confidence intervals reported. All tests formally reflected a superiority expectation. Type I error rates were controlled for by selecting and prioritizing tests, and therefore no statistical correction of multiple tests was carried out. No distributional assumptions were made. Covariate linearity was controlled using a likelihood ratio test of simple models with and without an added quadratic term. Baseline was defined as the third week of sickness absence, since all participants received sickness benefits, which are only available after at least four weeks of sick leave. Accordingly, no participants had returned to work before that period. The propensity score was estimated using a random forest, that is, an assumption-free tree-based ensemble model that captures non-linear associations well (i.e. interactions).

## Results

### Baseline

In this study, 151 participants gave written consent to receive real-world-INT. They were included from August 2018 to January 2019. From the group of 416 participants who in 2016–2018 were randomized RCT-INT, we identified and included 302 participants.

Baseline characteristics of the included participants are shown in Table 1. After matching, variables were balanced. Most participants were female (>70 %) and approx. 43 years of age (SD ~10–11). In the two years preceding index sick leave, participants had ~16 weeks (SD ~10) of sick leave and ~7–9 weeks (SD ~16–19) of unemployment. The average unemployment rate was 3.8% during the inclusion of the real-world-INT group and 4.2% regarding RCT-INT.

**Table 1:** Baseline characteristics.


	RCT-INT	REAL WORLD-INT	p-VALUE

*n* =	302	151	

*Sex (male)*	90 (29,8%)	48 (31,8%)	0.745

*Age*	43.17 (SD: 10,12)	42.72 (SD: 11,11)	0.676

*Diagnosis: Anxiety, other*	53 (17,5%)	19 (12,6%)	0.234

*Diagnosis: Anxiety, social*	14 (4,6%)	12 (7,9%)	

*Diagnosis: Exhaustion disorder*	86 (28,5%)	40 (26,5%)	

*Diagnosis: Depression, mild/moderate*	80 (26,5%)	48 (31,8%)	

*Diagnosis: Depression, severe*	26 (8,6%)	17 (11,3%)	

*Diagnosis: Stress*	43 (14,2%)	15 (9,9%)	

*Benefit history (no transfer in last 2 years)*	74.84 (24,4%)	71.69 (28,07%)	0.604

*Weeks on unemployment insurance (within 2 years)*	7.36 (SD: 16,63)	8.99 (SD: 19,41)	0.875

*Education (weeks within 2 years)*	0.52 (SD: 3,6)	0.83 (SD: 4,68)	0.570

*Sickness benefit (weeks within 2 years)*	15.52 (SD: 9,91)	16.54 (SD: 10,55)	0.162

*Parent leave (weeks within 2 years)*	3.30 (SD: 11,9)	3.26 (SD: 12,2)	0.854

*Subsidized employment (weeks within 2 years)*	0.24 (SD: 4,14)	0.53 (SD: 6,51)	0.617

*Unemployment benefit (weeks within 2 years)*	0.37 (SD: 3,25)	0.50 (SD: 3,67)	0.826


### Outcomes

**Figure 2:**
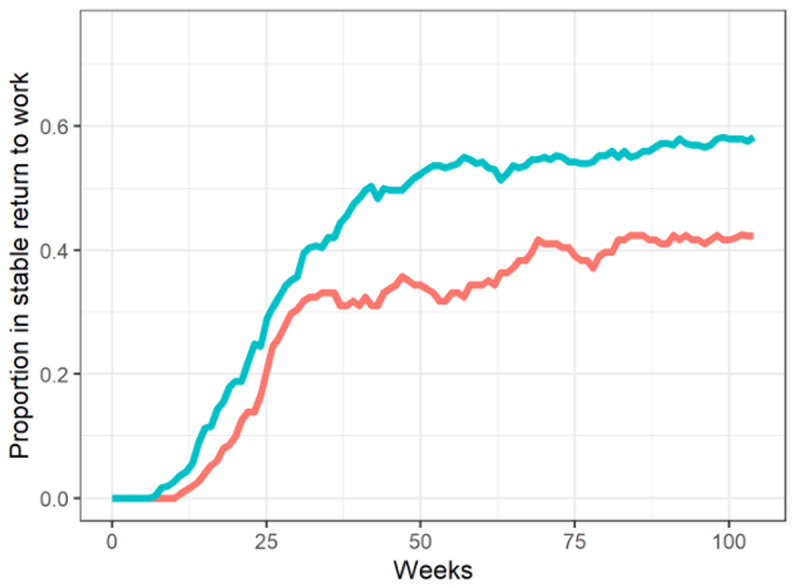
Proportion in stable work, per week. Red line: Real world-INT; Greenish line: RCT-INT.

On the primary outcome (at least 4 consecutive weeks in return to work within 12 months after baseline), 64.6% of the RCT-INT group had experienced RTW but only 48.3% in the real-world INT group (OR 0.51 [95%CI: 0.35–0.76], p = 0.001). The real world-INT group had a lower number of weeks in work (p < 0.001) at all follow-ups. Work status at 6-month follow-up was not statistically different between the two groups but at both 12- and 24-month follow-up, a smaller proportion of the real-world INT group were working, 42.3% vs. 58.3% in the RCT-INT group (p = 0.002). Similarly they have had less weeks in work since baseline at these two follow-ups, but not statistically sign. at 6-month follow-up. All outcome estimates are presented in Table 2, and Figure 2 shows the proportion in stable work per week after baseline.

**Table 2:** Vocational outcomes. ^+^: primary outcome. Ratio estimates are odds ratios for the binary outcomes and rate ratios for rate of weeks in employment.


OUTCOMES MEASURE	ESTIMATE	p-VALUE	RCT-INT (n = 302)	REAL WORLD-INT (n = 151)
	
	RATIOS (95% CI)		MEAN/N (SD/%)	MEAN/N (SD/%)

At any point within 24 months, *n (%)*	0.60 (0.38, 0.94)	0.027	235 (77.8%)	88 (67.7%)

**At any point within 12 months**, *n (%)*^+^	**0.51 (0.35, 0.76)**	**0.001**	**195 (64.6%)**	**73 (48.3%)**

At any point within 6 months, *n (%)*	0.73 (0.47, 1.12)	0.149	98 (32.5%)	39 (25.8%)

*Weeks within 24 months, mean (SD)*	0.70 (0.56, 0.84)	0.000	43.3 (SD: 33.2)	30.3 (SD: 29.6)

*Weeks within 12 months*, mean (SD)	0.68 (0.54, 0.85)	0.000	14.5 (SD: 14.3)	9.9 (SD: 12.6)

*Weeks within 6 months*, mean (SD)	0.56 (0.37, 0.82)	0.000	2.7 (SD: 4.9)	1.5 (SD: 3.4)

Status at 24 months, n (%)	0.53 (0.35, 0.80)	0.002	176 (58.3%)	55 (42.3%)

Status at 12 months, n (%)	0.43 (0.28, 0.64)	0.000	162 (53.6%)	50 (33.1%)

Status at 6 months, n (%)	0.73 (0.47, 1.14)	0.164	93 (30.8%)	37 (24.5%)


#### Sensitivity Analyses

No differences in results were observed between the main analysis and the sensitivity analyses, in which the 151 participants in the real world-INT group were matched to 151 participants in the RCT-INT group in the same municipality. Yet, on some measures the p-value was lower, thereby increasing the overall level of statistical significance in favour of the RCT-INT group’s outcomes. These results are found in the Results Supplement.

## Discussion

In this phase IV study, we tested the efficacy of The IBBIS Integrated Intervention (INT) when delivered in a real-world setting by comparing it to its effect when delivered in an RCT setting. To the authors’ belief this is the first phase 4 study in the area of return-to-work interventions for target populations of people on sick-leave with common mental disorders. On the primary outcome, we found a statistically significantly lower proportion of 48.3% had experienced return to work in the real-world-INT group, compared to 64.6% in the RCT-INT group (p = 0.001). Consistently this was the pattern among almost all other measures, with real-world-INT showing worse outcomes compared to RCT-INT.

We believe that most likely the greatest part of the difference is a true difference in causal effect between the two intervention delivery settings. In the randomized trials, we had observed moderate implementation issues, being a low fidelity particularly regarding the vocational rehabilitation intervention components, but a tendency of in rising fidelity over time. We therefore hypothesized that the effect could be similar in the real-world setting, due to fidelity perhaps reaching a level to at least be sufficient to equal the effect in the RCT setting. Contrary to this hypothesis, we saw that vocational outcomes of real-world INT were significantly worse across almost all measures and different periods of follow-up. We believe that a substantial part of the negative outcomes reflects the difference between interventions in real-world vs. RCT settings that is often referred to as the difference between effectiveness and efficacy [33]. One possible explanation could be that the real-word setting implementation was relatively poorer – a tendency also observed in other comparisons of RCTs and real-world settings [33]. During the RCT, managerial focus on protocol adherence was greater to ensure protocol fidelity and, subsequently, internal validity, but the results in this study support the suspicion that the intervention might be rather unfeasible.

We cannot completely rule out that unobserved between-group differences confounded the results. It is, for example, possible that the level of disorder was higher in the real-world group, causing worse outcomes. While we tried to handle this potential confounder by matching diagnoses, levels of illness could still vary within diagnostic groups because the municipal body had different incentives for referring potential participants during recruitment to the real-world study (*after* the RCTs). Other studies have previously suggested that RCT participants and real-world individuals may differ [34]. Societal factors that change over time, like e.g. employment rates could explain some of the findings through confounding, but it is not very likely. During observation of the real-world group, the average baseline unemployment rate was lower, and one would therefore have expected *increased* return-to-work rates among sickness absentees rather than the *decreased* rates we observed [35]. Similarly, sickness benefit legislation was reformed some years prior to the study, and that could influence the general return to work rates differently in the two time periods the two study groups were compared. Yet, the RCTs took place during the gradual implementation of this reform, from 2016 to 2019, and we observed that return-to-work rates after sick leave tended to *increase* over time, so this cannot explain the decrease in return-to-work rates seen in this study. Finally, sensitivity analyses were performed to adjust for municipality, but though the sample size of the control group was halved, statistical significance increased, making it more likely that results represented a true difference.

### Strengths and limitations

The major strength of this study is the ability to track all participants in a highly consistent manner with few-to-no missing data. Given the comprehensive and precise Danish registers, individual observations are probably quite accurate. However, the use of register-based data also has certain limitations, most notably the fact that the available information only describes utilization of sickness benefits and not level of disease. Several other studies have shown that the utilization of sickness benefits is influenced by a range of contextual factors other than level of disease, for example the degree of employment protection in a country [36] and the level of sick leave benefit control [37]. This study is limited by the fact that the two study groups were separated in time, and we cannot exclude the possibility that time is a confounder, not least since contextual factors might have changed between the study periods. However, as discussed above, this effect alone would most likely produce the opposite of what we have found. Another strength is that the study was publicly pre-registered before any analysis was performed, thus reducing the risk of Type 1 errors. While not adjusting for multiple tests could be a limitation, it is negligible in this study since all findings of highly statistically significant differences favour only one of the interventions. Also, all tests support the same overall hypothesis of treatment effects on vocational recovery.

## Conclusion

This study has a number of implications. First and foremost, it highlights that INT is either non-feasible or ineffective for common mental disorders on a group level. While the original IBBIS Trial targeting people with anxiety and depression tended to show some positive effect in the group of people with anxiety, this effect was not seen consistently across outcome measures, and this study casts further doubt on the effectiveness of the intervention, specifically when these are translated into real-world setting. Assuming that interventions are usually implemented with higher protocol fidelity in RCTs than in real-world settings, this study confirms that the efficacy of a complex intervention like INT requires a high level of managerial control, as previously documented in the process evaluation of the original RCTs [18]. While INT may have positive effects that are not observed in this study, for example on well-being, implementing INT in a real-world setting like it was here will most likely not improve vocational outcomes for the common mental disorder population as a whole.

## Additional File

The additional file for this article can be found as follows:

10.5334/ijic.7562.s1 Results Supplement. This supplement contains results of the sensitivity analysis.
