# Supplementary material for: The Danish Ibbis Trials for Sickness Absentees with Common Mental Disorders: A Phase 4 Prospective Study Comparing Randomized Trial and Real-World Data
Source: Int J Integr Care. 2024 Jul 26;24(3):10. doi: 10.5334/ijic.7562 (PMC11276403; doi:10.5334/ijic.7562)
Supplement: Results Supplement. — This supplement contains results of the sensitivity analysis. [file ijic-24-3-7562-s1.pdf]

RESULTS SUPPLEMENT

Regarding the paper: *The Danish IBBIS Trials for sickness absentees with common mental disorders: A Phase 4 prospective study comparing randomized trial and real-world data*

This supplement contains results of the sensitivity analysis, described under “Methods” in the main paper. In the control group in this analysis, only participants from Copenhagen Municipality have been selected. The intervention group is the same (and is also only participants from Copenhagen Municipality).

The format follows the structure of tables and figures of the main analyses, corresponding to Table 1, Table 2 and Figure 1 in the main paper.

	**RCT-INT**	**Real world-INT**	**p-value**
n=	151	151	
Sex (male)	44 (29,1%)	48 (31,8%)	0,708
Age	42,66 (SD: 10,47)	42,72 (SD: 11,11)	0,963
Diagnosis: Anxiety, other	29 (19,2%)	19 (12,6%)	0,057
Diagnosis: Anxiety, social	5 (3,3%)	12 (7,9%)	
Diagnosis: Exhaustion disorder	50 (33,1%)	40 (26,5%)	
Diagnosis: Depression, mild/moderate	30 (19,9%)	48 (31,8%)	
Diagnosis: Depression, severe	18 (11,9%)	17 (11,3%)	
Diagnosis: Stress	19 (12,6%)	15 (9,9%)	
Benefit history (no transfer in last 2 years)	4,21 (SD: 0,11)	3,82 (SD: 0,04)	0,999
Weeks on unemployment insurance (within 2 years)	7 (4,6%)	13 (8,6%)	0,835
Education (weeks within 2 years)	18 (11,9%)	20 (13,2%)	0,175
Sickness benefit (weeks within 2 years)	14 (9,3%)	12 (7,9%)	0,742
Parent leave (weeks within 2 years)	25 (16,6%)	21 (13,9%)	0,558
Subsidized employment (weeks within 2 years)	7 (4,6%)	6 (4%)	0,569
Unemployment benefit (weeks within 2 years)	14 (9,3%)	13 (8,6%)	0,746

Table 1: Baseline characteristics

Measures of work	Estimate	p-value	RCT-INT	Real world-INT
n=			151	151
At any point within 24 months	0.586	0,066	118 (78,1%)	88 (67,7%)
**At any point within 12 months (primary outcome)**	**0.450***	**0,001**	**102 (67,5%)**	**73 (48,3%)**
At any point within 6 months	0.663	0,132	52 (34,4%)	39 (25,8%)
Weeks within 24 months	0.698*	0,001	43,4 (SD: 32,5)	30,3 (SD: 29,6)
Weeks within 12 months	0.640*	0,000	15,5 (SD: 14,6)	9,9 (SD: 12,6)
Weeks within 6 months	0.484*	0,028	3,1 (SD: 5,3)	1,5 (SD: 3,4)
Status at 24 months	0.601*	0,046	83 (55%)	55 (42,3%)
Status at 12 months	0.395*	0,000	84 (55,6%)	50 (33,1%)
Status at 6 months	0.696	0,201	48 (31,8%)	37 (24,5%)

*Table 2: Vocational outcomes; *: p < 0.05*

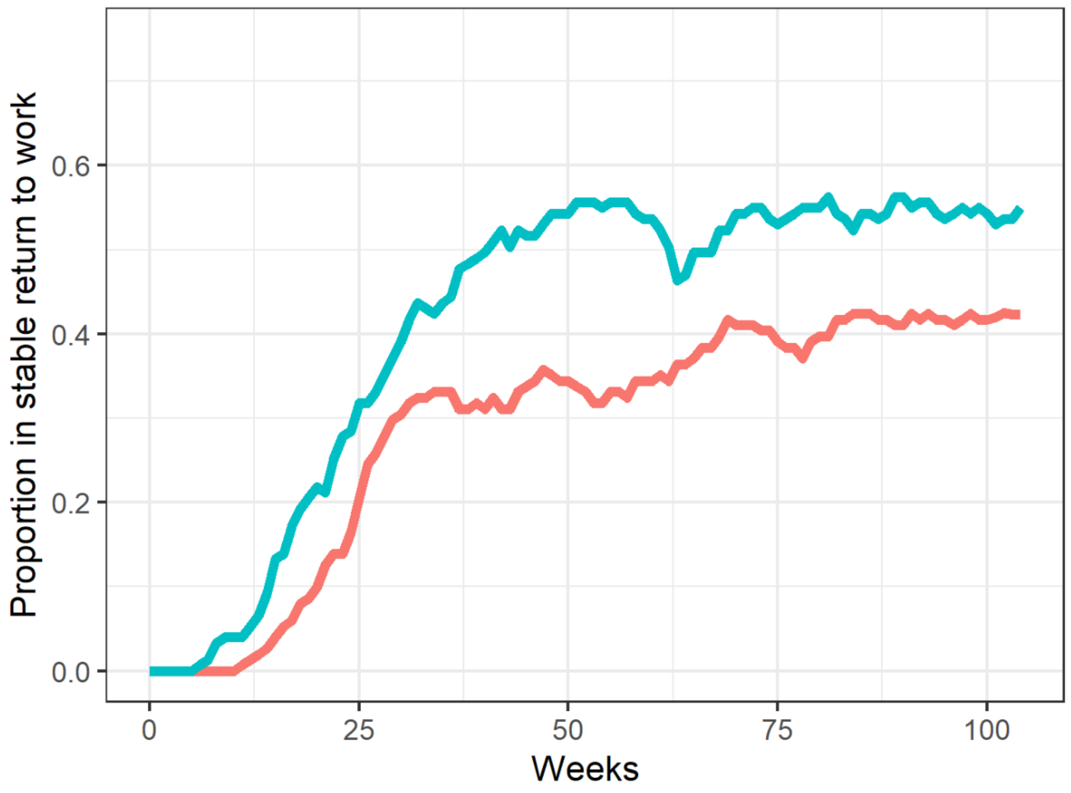

Figure 1: Proportion in stable work, per week. Red line: Real world-INT; Greenish line: RCT-INT
